# Associação entre sedentarismo e nível socioeconômico em adolescentes** ***

**DOI:** 10.15649/cuidarte.2082

**Published:** 2022-08-14

**Authors:** Fabiangelo de Moura Carlos, Paulo Henrique Alves de Sousa, Cezenário Gonçalves Campos, Joel Alves Lamounier, Wendell Costa Bila, Márcia Christina Caetano Romano

**Affiliations:** 1 Universidade Federal de São João Del Rei (CCO), Divinópolis, MG, Brasil. Email: fabiangelomc@gmail.com Universidade Federal de São João del-Rei Universidade Federal de São João Del Rei Divinópolis MG Brazil fabiangelomc@gmail.com; 2 Universidade Federal de São João Del Rei (CCO), Divinópolis, MG, Brasil. Email: spaulohenrique@hotmail. com Universidade Federal de São João del-Rei Universidade Federal de São João Del Rei Divinópolis MG Brazil spaulohenrique@hotmail. com; 3 Universidade Federal de São João Del Rei (CCO), Divinópolis, MG, Brasil. Email: cezenario@yahoo.com.br Universidade Federal de São João del-Rei Universidade Federal de São João Del Rei Divinópolis MG Brazil cezenario@yahoo.com.br; 4 Universidade Federal de São João Del Rei (CDB), São João Del Rei, MG, Brasil. Email: lamounierjoel@gmail.com Universidade Federal de São João del-Rei Universidade Federal de São João Del Rei São João Del Rei MG Brazil lamounierjoel@gmail.com; 5 Universidade Federal de São João Del Rei (CCO), Divinópolis, MG, Brasil. Email: wendellbila1@gmail.com Universidade Federal de São João del-Rei Universidade Federal de São João Del Rei Divinópolis MG Brazil wendellbila1@gmail.com; 6 Universidade Federal de São João Del Rei (CCO), Divinópolis, MG, Brasil. Email: marciachristinacs@gmail.com Universidade Federal de São João del-Rei Universidade Federal de São João Del Rei Divinópolis MG Brazil marciachristinacs@gmail.com

**Keywords:** Adolescente, Comportamento Sedentário, Classe Social, Promoção da Saúde Escolar, Adolescent, Sedentary Behavior, Social Class, School Health Services, Adolescente, Conducta Sedentaria, Clase Social, Promoción de la salud escolar

## Abstract

**Introdução:**

O sedentarismo em adolescentes contribui para a ocorrência de diferentes doenças, sendo relevante investigar sobre fatores associados.

**Objetivo:**

Analisar a associação entre sedentarismo e nível socioeconômico em adolescentes de escolas públicas.

**Materiais e Métodos:**

Estudo transversal, realizado com 347 adolescentes matriculados em escolas públicas do ensino médio do município de Divinópolis, Minas Gerais. A coleta de dados ocorreu no ano de 2017. O sedentarismo foi avaliado utilizando-se o *International Physical Activity Questionnaire* e o nível socioeconômico pelo critério da Associação Brasileira de Empresas de Pesquisa. Foi realizada estatística descritiva e analítica através de modelo de regressão logística multivariada.

**Resultados:**

Participaram da investigação 347 adolescentes. A média de idade do grupo foi de 16,4 ± 1,0 anos. Os indivíduos caracterizados como sedentários constituíram 38,9% da amostra, sendo que, destes, 66,7% eram do sexo feminino. Possuir maior nível socioeconômico diminui a probabilidade de ser sedentário (OR=0.235; 95%IC: 0,069-0,803; p=0.021), assim como ser estudante das escolas públicas da região sudoeste aumenta essa chance(OR=2,68; 95%IC:1,370-5,239; p=0,04 ).

**Discussão:**

Os motivos pelos quais as condições socioeconômicas podem influenciar o sedentarismo são variados. A ausência de espaços públicos pode contribuir para a elevação do sedentarismo em adolescentes com menor nível socioeconômico.

**Conclusão:**

Esta investigação sinaliza a importância de investimentos públicos em políticas de estímulo à prática de atividade física para os adolescentes, em especial para os do sexo feminino e de menor nível socioeconômico.

## Abstract (en)

**Introduction:**

A sedentary lifestyle in adolescents contributes to the occurrence of different diseases, making it relevant to investigate associated factors.

**Objective:**

To analyze the association between sedentary lifestyle and socioeconomic status in adolescents from public schools.

**Materials and Methods:**

Cross-sectional study, carried out with 347 adolescents enrolled in public high schools in the city of Divinópolis, Minas Gerais. Data collection took place in 2017. Sedentary lifestyle was assessed using the International Physical Activity Questionnaire and socioeconomic status using the criteria of the Brazilian Association of Research Companies. Descriptive and analytical statistics were performed using a multivariate logistic regression model.

**Results:**

347 adolescents participated in the investigation. The mean age of the group was 16.4 ± 1.0 years. Individuals characterized as sedentary constituted 38.9% of the sample, of which 66.7% were female. Having a higher socioeconomic level decreases the probability of being sedentary (OR=0.235; 95%IC: 0,069-0,803; p=0.021), just as being a student in public schools in the southwest region increases this chance (OR=2,68; 95%IC:1,370-5,239; p=0,04).

**Discussion:**

The reasons why socioeconomic conditions can influence a sedentary lifestyle are varied. The absence of public spaces can contribute to the increase in sedentary lifestyles in adolescents with lower socioeconomic status.

**Conclusion:**

This investigation highlights the importance of public investments in policies to encourage the practice of physical activity for adolescents, especially for females and lower socioeconomic status.

## Resumen

**Introducción:**

El sedentarismo en los adolescentes contribuye a la ocurrencia de diferentes enfermedades, por lo que es relevante investigar los factores asociados.

**Objetivo:**

Analizar la asociación entre sedentarismo y nivel socioeconómico en adolescentes de escuelas públicas.

**Materiales y Métodos:**

Estudio transversal, realizado con 347 adolescentes matriculados en escuelas secundarias públicas de la ciudad de Divinópolis, Minas Gerais. La recolección de datos tuvo lugar en 2017. El estilo de vida sedentario se evaluó mediante el Cuestionario Internacional de Actividad Física y el nivel socioeconómico según los criterios de la Asociación Brasileña de Empresas de Investigación. La estadística descriptiva y analítica se realizó mediante un modelo de regresión logística multivariante.

**Resultados:**

347 adolescentes participaron en la investigación. La edad media del grupo fue de 16,4 ± 1,0 años. Los individuos caracterizados como sedentarios constituyeron el 38,9% de la muestra, de los cuales el 66,7% eran mujeres. Tener un nivel socioeconómico más alto disminuye la probabilidad de ser sedentario (OR=0.235; 95%IC: 0,069-0,803; p=0.021), al igual que ser alumno de escuelas públicas de la región suroeste aumenta esta posibilidad (OR=2,68; 95%IC:1,370-5,239; p=0,04).

**Discusión:**

Las razones por las que las condiciones socioeconómicas pueden influir en un estilo de vida sedentario son variadas. La ausencia de espacios públicos puede contribuir al aumento de estilos de vida sedentarios en adolescentes de menor nivel socioeconómico.

**Conclusión:**

Esta investigación destaca la importancia de la inversión pública en políticas para incentivar la práctica de actividad física en adolescentes, especialmente en mujeres y estrato socioeconómico más bajo.

## Introdução

O sedentarismo é considerado um sério problema de saúde pública e um dos principais fatores de risco de morte no mundo. No Brasil, aproximadamente 40% da população geral é sedentária. Cerca de 3,2 milhões de pessoas morrem a cada ano em decorrência de doenças oriundas da inatividade física, como obesidade, doenças cardiovasculares, diabetes *mellitus* tipo II, depressão e alguns tipos de câncer^1^. Entre adolescentes e jovens tal situação é responsável ainda por ocasionar baixa autoestima, problemas relacionados ao comportamento social, além de desempenho acadêmico insuficiente^2^.

Estudo realizado em escolas de 146 países, incluindo 1,6 milhão de alunos com idades entre 11 e 17 anos, cerca de 81% dos adolescentes não se movimentam de maneira suficiente. Eles praticam menos de uma hora por dia de atividade física, o que pode acarretar sérios danos à saúde. Chama a atenção o fato de que o problema é maior entre as meninas^3^.

Existe um consenso sobre os baixos níveis de atividade física entre adolescentes nos diversos países^4^.No Brasil, em 2015, também se comprovou essa realidade, pois a Pesquisa Nacional de Saúde do Escolar (PENSE) mostrou que a maioria dos adolescentes brasileiros é sedentária. A prevalência de sedentarismo no território nacional varia de 60% na região Norte a 70% na região Sudeste^5^.

Estudo com adolescentes de diversos países, realizado entre os anos 2001 e 2016, destacou que o Brasil alcançou prevalência de 83,6% de sedentarismo na população na faixa etária de 10 a 19 anos. Resultados dessa investigação apontaram também que as maiores diferenças foram registradas nos Estados Unidos da América e na Irlanda^6^. Os estudos apresentados demonstram que parece haver uma pandemia de sedentarismo entre os adolescentes^4^^,^^6^.

Os estudos demonstram que parece haver um perfil para os adolescentes sedentários. A desigualdade econômica, o consumo de alimentos não saudáveis, o excesso de peso, a escolaridade dos pais, a renda familiar e a ocupação dos pais são variáveis que podem estar associadas ao sedentarismo^3^^,^^4^.

O sedentarismo pode ser influenciado por vários elementos, como cultura, a vida moderna e suas demandas, bem como o nível de escolaridade e nível socioeconômico (NSE)^7^. O nível socioeconômico destaca-se neste conjunto, pois acredita-se que exerça forte influência nas atitudes pessoais, experiências, bem como na exposição a diferentes fatores de risco à saúde^8^.

Autores acreditam que a condição socioeconômica interfere na prática de atividade física, podendo ser um facilitador ou uma barreira^9^. Nessa perspectiva, pesquisadores afirmam que ter acesso à prática de atividade física e de esportes relaciona-se com o fato de a família ter condições financeiras para ofertar tais oportunidades^10^. Outros investigadores contradizem essa afirmativa, pontuando que adolescentes de países de alta renda tendem a gastar mais tempo em comportamento sedentário em relação àqueles de menor renda^11^. Nessa direção, há uma lacuna no conhecimento e ausência de consenso acerca da relação entre nível socioeconômico e sedentarismo entre adolescentes.

Considerando as diversas implicações do sedentarismo para a saúde do adolescente^2^, torna-se cada vez mais relevante investigar sobre essa temática, identificando elementos que possam estar associados a esse fenômeno. Acredita-se que a presente investigação poderá contribuir para o mapeamento do sedentarismo entre adolescentes escolares do ensino médio de Divinó-polis/ MG, além de trazer indicadores para potencializar e justificar maiores investimentos em programas para a promoção da atividade física no município. O objetivo desse estudo é analisar a associação entre sedentarismo e nível socioeconômico em adolescentes de escolas públicas.

## Materiales y Métodos

O presente trabalho é produto de dissertação de mestrado do Programa de Pós-Graduação em Enfermagem da Universidade Federal de São João Del-Rei, Campus Centro-Oeste, UFSJ-CCO.Os dados da pesquisa é parte de um projeto maior intitulado “Composição corporal e avaliações genéticas em adolescentes e jovens com sobrepeso e obesos, submetidos a programas diferenciados de atividade física”, pesquisa realizada no município de Divinópolis, Minas Gerais, no ano de 2017 e aprovado pelo Comitê de Ética e Pesquisa CEPES/ UFSJ-CCO, parecer número CAAE: 61665716.9.0000.5545. Em 2020, foram realizadas análises estatísticas com intuito de responder ao objetivo deste trabalho com a utilização do banco de dados vinculado ao Mendeley^12^. Deste modo justifica-se o espaço de tempo entre a coleta de dados e a submissão dos atuais resultados.

Trata-se de estudo de base escolar, de abordagem quantitativa, do tipo transversal, realizado nas 18 escolas estaduais de nível médio de ensino do Município de Divinópolis, MG. O desenho do estudo seguiu as normas do “Strengthening the Reporting of Observational Studies in Epidemiology statement” (STROBE)^13^.

A população elegível do estudo constituiu de 7.001 adolescentes de 15 a 19 anos de idade (neste estudo a faixa etária utilizada referente à adolescência é a padronizada pela Organização Mundial de Saúde)1, regularmente matriculados nas escolas estaduais de nível médio de Divinópolis. O município dispõe de dezoito escolas estaduais localizadas no território urbano que oferecem o ensino médio regular. Segundo dados do IBGE, a cidade tem aproximadamente 233 mil habitantes e possui Índice de Desenvolvimento Humano (IDH) de 0,7613.

Foi desenvolvido um processo de amostragem aleatória simples. Todas as dezoito escolas estaduais foram convidadas, aceitaram participar e contribuíram na amostra com a quantidade de alunos proporcionalmente à sua equivalência no universo amostral e proporcionalmente à faixa etária dos 15 aos 19 anos. O volume de alunos sorteado nas respectivas turmas em cada escola também seguiu a sua respectiva proporcionalidade, de maneira que todos os estudantes da cidade tiveram a mesma chance de serem sorteados para participar da pesquisa, garantindose assim a plena representatividade da amostra, observando-se os critérios de inclusão e exclusão.

Assim, nossa amostra do estudo foi constituída de 347 adolescentes. Para o cálculo amostral, utilizou-se o programa estatístico de domínio público *Open Epi* 3.01 e considerou-se a população descrita de 7.001 para um nível de confiança de 95%, precisão absoluta de 5% e proporção de inatividade física entre os adolescentes dessa faixa etária de 35,9%, segundo prevalência encontrada em estudo entre adolescentes escolares da mesma faixa etária em um município do Estado de Minas Gerais^14^.

Foram adotados como critérios de inclusão: adolescentes de ambos os sexos na faixa etária de 15 a 19 anos, indivíduos matriculados no ensino médio da rede pública estadual de ensino aptos a frequentar as aulas de educação física escolar e atividades físicas.

Foram considerados critérios de exclusão: relatos de diagnóstico de doenças cardiológicas, reumatológicas, limitações ortopédicas, respiratórias, doenças do sistema nervoso central e do sistema endócrino, ou qualquer outra doença que pudesse interferir na prática de atividade física.

Os pais e/ou responsáveis pelos alunos que atenderam aos critérios de inclusão foram informados sobre os objetivos da pesquisa e procedimentos a serem realizados. Foi feita solicitação da assinatura do Termo de Consentimento Livre e Esclarecido TCLE aos participantes com idade igual ou maior que 18 anos idade. No caso dos participantes menores de 18 anos de idade, a assinatura do TCLE foi coletada dos seus pais/responsáveis, além do Termo de assentimento aplicado aos menores de 18 anos de idade e assinado por eles.

A coleta de dados foi realizada pelos autores deste artigo e constituiu-se de avaliação diagnóstica, subsidiando a identificação da idade, sexo, raça, peso, estatura, índice de massa corporal (IMC), nível socioeconômico e nível de atividade física.

Foi utilizado o questionário da Associação Brasileira de Empresas e Estatística (ABEP) para o conhecimento do nível socioeconômico. Esse instrumento faz a mensuração do nível sócio econômico das famílias e se baseia no cômputo dos bens existentes no domicílio, cujas categorias podem variar de A até E, de acordo com a pontuação obtida, sendo A o maior nível e E o menor. O questionário é validado para população brasileira^15^.

O nível de atividade física dos adolescentes participantes do estudo foi mensurado pelo *International Physical Activity Questionnaire* (IPAQ), versão curta, Guedes e colaboradores reproduziram e validaram o instrumento em adolescentes brasileiros. Para categorização da atividade física habitual, foram considerados sedentários os participantes classificados em irregularmente ativos e sedentários, assim como considerados não-sedentários aqueles adolescentes classificados como muito ativos e ativos, baseado no consenso proposto pelo Centro Laboratório de Estudos de Aptidão Física de São Caetano do Sul^16^.

A avaliação antropométrica (peso e estatura) foi efetuada de acordo com métodos recomendados pela Organização Mundial da Saúde (OMS) e pelo Ministério da Saúde. Os dados foram coletados em triplicatas e, em seguida, a média aritmética foi calculada. A aferição do peso foi realizada em balança eletrônica digital da marca Tanita®, modelo BF-683 W, com capacidade máxima de 150 quilogramas (kg) e precisão de 100 gramas (g), conforme as técnicas preconizadas por JELLIFE^17^. As mensurações foram realizadas com o adolescente em posição ortostática, descalço, com os membros superiores estendidos ao longo do corpo e com o olhar fixado no horizonte, de modo a evitar oscilações na leitura da medida^18^^-^^19^. A medida da estatura foi aferida utilizando-se antropômetro vertical móvel, da marca Alturaexata®, com graduação em centímetros (cm) até 2,13 metros e precisão de 0,1cm.

Foi calculado o índice de massa corporal (IMC) por intermédio da razão entre a massa corporal pela estatura elevada ao quadrado (kg/m2). Todo o processo de avaliação antropométrica foi realizado em local reservado, de forma individual. Foi utilizado o programa *WHO Anthroplus®*, disponibilizado pela OMS, para avaliação e categorização do estado nutricional dos adolescentes, especialmente pela interpretação do escore Z de IMC por idade, classificando os adolescentes em magreza acentuada, magreza, eutrofia, sobrepeso, obesidade e obesidade grave^18^.

Os dados utilizados nas avaliações foram digitados e organizados no modelo planilha do *EpiData®*, de domínio público, com dupla entrada de dados, compondo o banco de dados para o estudo. A análise dos dados utilizou o programa estatístico *Statistical Package for the Social Sciences (SPSS),* versão 20.0.

Foram utilizadas técnicas de estatística descritiva e analítica. Os resultados descritivos foram obtidos por meio das medidas de tendência central e dispersão, frequências absolutas e relativas, assim como as prevalências de sedentarismo de forma regionalizada no município. O teste de *Shapiro-Wilk* disponibilizado pelo programa estatístico foi utilizado para verificação da normalidade da distribuição dos valores das variáveis contínuas.

A estatística analítica utilizou análises de regressão logística/qui-quadrado para verificação de associação entre a variável dependente e as possíveis variáveis independentes/ explicativas. Foi observado o intervalo de confiança de 95%, ou seja, nível de significância de 5% (α=0,05), p-valor = 0,05.

## Resultados

Participaram da investigação 347 adolescentes. A média de idade do grupo foi de 16,4 ± 1,0 anos. Os indivíduos caracterizados como sedentários constituíram 38,9% da amostra, sendo que, destes, 66,7% eram do sexo feminino. A análise descritiva está sumarizada na Tabela 1.

**Tabela 1:** Distribuição dos participantes do estudo conforme hábitos de vida, características sociodemográficas e nutricionais, Divinópolis, Minas Gerais. 2020*. N=347

Variável	Fem	Masc	Total
Sedentarismo			
Sim	90 (25,9%)	45 (13,0%)	135 (38,9%)
Não	119 (34,3%)	93 (26,8%)	212 (61,1%)
Total	209 (60,2%)	138 (39,8%)	347 (100,0%)
Nível socioeconômico			
A	7 (2,0%)	11 (3,2%)	18 (5,2%)
B1	16 (4,7%)	18 (5,2%)	34 (9,9%)
B2	54 (15,7%)	41 (11,9%)	95 (27,6%)
C1	64 (18,6%)	35 (10,2%)	99 (28,8%)
C2	47 (13,7%)	26 (7,6%)	73 (21,2%)
D -E	21 (6,1%)	4 (1,2%)	25 (7,3%)
Idade			
15	37 (10,7%)	30 (8,6%)	67 (19,3%)
16	82 (23,6%)	44 (12,7%)	126 (36,3%)
17	56 (16,1%)	41 (11,8%)	97 (28,0%)
18	29 (8,4%)	18 (5,2%)	47 (13,5%)
19	5 (1,4%)	5 (1,4%)	10 (2,9%)
Estado nutricional			
Magreza acentuada	1 (0,3%)	1 (0,3%)	2 (0,6%)
Magreza	8 (2,3%)	7 (2,0%)	15 (4,3%)
Eutrofia	168 (48,4%)	90 (25,9%)	258 (74,4%)
Sobrepeso	23 (6,6%)	26 (7,5%)	49 (14,1%)
Obesidade	8 (2,3%)	11 (3,2%)	19 (5,5%)
Obesidade grave	1 (0,3%)	3 (0,9%)	4 (1,2%)

Os dados foram coletados no ano de 2017. Contudo as análises estatísticas que respondem o objetivo desta pesquisa foram realizadas em 2020. Fonte: Dados da pesquisa

Abaixo, a Tabela 2 mostra as diferenças de prevalência de sedentarismo entre as regiões do município de Divinópolis. Foi verificada diferença estatisticamente significativa entre as prevalências de sedentarismo das regiões nordeste (35,7%) e oeste (30,0%).

**Tabela 2:** Distribuição dos participantes de estudo conforme o sedentarismo e as regiões de planejamento do Município. Divinópolis/Minas Gerais, 2020*. N=347

	Noroeste	Nordeste	Sudoeste	Sudeste	Central	Oeste
Sedentarismo	23(35,9%)	5(35,7%)	31(56,4%)	38(40,4%)	35(31,8%)	3(30,0%)
Noroeste	X	0,622	0,217	0,592	0,708	0,591
Nordeste	0,622	X	0,455	0,258	0,467	0,023*
Sudoeste	0,217	0,455	X	0,426	0,708	0,521
Sudeste	0,592	0,258	0,426	X	0,183	0,580
Central	0,708	0,467	0,708	0,183	X	0,700
Oeste	0,591	0,023*	0,521	0,580	0,700	X

*Os dados foram coletados no ano de 2017. Contudo as análises estatísticas que respondem o objetivo desta pesquisa foram realizadas em 2020. Fonte: Dados da pesquisa

A análise de regressão logística multivariada relativa ao sedentarismo e suas variáveis associadas está apresentada na Tabela 3. Houve associação significativa entre sedentarismo e o pertencimento à classe social B1 (OR=0,235 p=0,021), bem como estar matriculado em escolas localizadas na região sudoeste do município (OR=2,680 p=0,04), ou seja, indivíduos da classe B1 têm menos chance de pertencer ao grupo sedentário. Além disso, os estudantes de escolas da região sudoeste apresentaram quase três vezes mais chance de estarem sedentários, quando comparados aos da região central.

**Tabela 3:** Análise de regressão logística multivariada entre o sedentarismo e as variáveis explicativas para o modelo teórico. Divinópolis, Minas Gerais, 2020*. N=347

Variável Nível socioeconômico	OR	95% IC	p-valor
A**	1,000	-	-
B1	0,235	0,069-0,803	0,021*
B2	0,493	0,177-1,375	0,177
C1	0,507	0,182-1,414	0,194
C2	0,373	0,129-1,079	0,069
D -E	0,722	0,209-2,494	0,606
Idade (anos completos)			
15**	1,000	-	-
16	1,207	0,644-2,263	0,558
17	1,837	0,958-3,5 23	0,067
18	1,026	0,463-2,271	0,950
19	0,896	0,210-3,835	0,883
Raça auto declaradas			
Branca**	1,000	-	-
Parda	0,850	0,524-1,378	0,510
Negra	1,036	0,530-2,026	0,917
Oriental	-	-	-
Indígena	1,808	0,460-7,111	0,397
Estado nutricional	1,276	0,743-2,189	0,377
Regionalização			
Central**	1,000	-	-
Noroeste	1,236	0,643-2,375	0,525
Nordeste	1,142	0,354-3,678	0,824
Sudoeste	2,680	1,370-5,239	0,004*
Sudeste	1,501	0,841-2,678	0,169
Oeste	0,884	0,214-3,645	0,865

*Os dados foram coletados no ano de 2017. Contudo as análises estatísticas que respondem o objetivo desta pesquisa foram realizadas em 2020. Fonte: Dados da pesquisa

## Discussão

O sedentarismo de 38,9% encontrado no presente estudo assemelha-se a outros achados, como o estudo realizado com 960 adolescentes entre 15 e 18 anos, na cidade brasileira de Pelotas, conduzido por Oehlschlaeger et al (2004)^20^, que encontrou uma prevalência de 39%.

Os resultados indicaram que adolescentes da classe B1 têm menos chance de serem sedentários quando comparados aos da classe socioeconômica A, desta forma, pertencer a uma classe socioeconômica mais baixa diminui a probabilidade de adolescentes serem sedentários. Tal fato também foi evidenciado por Oehlschlaeger et al (2004), que afirmaram que adolescentes das classes sociais mais baixas em uma cidade brasileira apresentaram vez mais chances de estarem sedentários 1,35 (IC 95%1,06-1,72)^20^.

Também foi identificado que adolescentes matriculados em escolas localizadas na região sudoeste do município tiveram mais chance de serem sedentários. Em seu estudo com estudantes de ensino médio de João Pessoa, Brasil, de Lucena et al (2015) identificaram que o tempo de tela foi alto (79.5%; 95%CI: 78.1-81.1) e variava de acordo com as características sociodemográficas, sendo que os adolescentes de classe socioeconômica mais elevada possuíam mais chances de exposição ao tempo de tela (OR=1.49; 95%CI: 1.21-1.84)^21^.

Os motivos pelos quais as condições socioeconômicas podem influenciar o sedentarismo são variados. Quando o sedentarismo é mais prevalente em classes mais favorecidas, autores justificam com o argumento de que indivíduos de extratos sociais mais baixos tendem a ter atividades menos sedentárias, como por exemplo, as laborais que exigem esforço físico, diminuindo a inatividade física. Mesmo não sendo atividades orientadas e muitas das vezes adequadas para faixa etária, não deixam de mobilizar o corpo e gerar gasto energético significativo^21^.

Outro fator que pode estar envolvido com o fato de adolescentes de classes mais favorecidas economicamente estarem mais sedentários é o acesso a tecnologias e consequente elevado tempo de tela. Estudiosos têm apresentado a relação do nível de atividade física e aumento do tempo de tela com diversas variáveis biológicas, sociodemográficas e psicossociais em adolescentes^22^. O efeito da classe econômica pôde ser percebido em um estudo avaliando 92 estudantes do ensino médio, onde há menor chance do grupo de adolescentes trabalhadores assistirem a TV excessivamente quando comparado ao grupo não trabalhador (p=0,013), caracterizando então um comportamento para o sedentarismo maior nas classes econômicas mais elevadas^23^.

Seguindo esse mesmo raciocínio, famílias de maior poder aquisitivo podem proporcionar a seus filhos o acesso a opções de lazer com caráter mais sedentário, como videogames, computadores e suas variações, como *tablets* e telefones celulares com maior acesso a internet^24^.

Contrapondo nossos resultados, Christofoletti*et al.*, em seu estudo com 484 estudantes adolescentes da cidade de Rio Claro (SP) não encontraram associação entre as classes econômicas e estado de comportamento sedentário total. Nessa investigação, o sedentarismo foi avaliado por meio do Questionário de Comportamento Sedentário com validade no Brasil para a população adulta e não para adolescentes como em nosso estudo, o que pode justificar a diferença^25^.

Entretanto, em seu estudo com 28.031 adolescentes da União Europeia, Moreno-Llamas et al (2020) apresentaram que a classe social baixa era mais propensa a ser ativa em comparação com a classe social alta (OR 0,52; IC95%: 0,47-0,58), e a mesma situação ocorria com a classe social média (OR 0,71; IC95%: 0,64-0,79)^26^.

Outro aspecto relevante em nossa investigação é que estudantes da região sudoeste do município têm mais chance de serem sedentários. Sabe-se que em regiões onde há melhor infraestrutura urbana, segurança, iluminação, presença de espaços adequados para exercícios físicos, existe maior motivação para a prática de esportes e atividades físicas e consequente baixo índice de sedentarismo^27^.

Aspectos relacionados à privação de oportunidades derivada de local de moradia (como renda, educação, emprego e saúde) foram analisados no estudo realizado por Combs et al (2013). Os autores concluíram que o nível socioeconômico mais baixo está associado a níveis mais altos de visualização de TV (p<0,05)^28^.

A região Sudoeste de Divinópolis está em largo processo de desenvolvimento e é onde se encontram muitas das principais obras da cidade, mas não consta de opções de espaços públicos adequados para a prática de atividades físicas. É encontrado nessa região um elevado número de academias, no entanto, de acordo com o estudo desenvolvido por Mendonça et al (2018)^29^, apesar de os exercícios físicos e esportes serem as atividades de preferências entre os adolescentes (21,8% e 8,1%, respectivamente), estas requerem recursos financeiros para sua prática, o que acaba possivelmente por não atender grande parte dos jovens desta região. Portanto, pode-se entender que questões como infraestrutura dos bairros e engajamento dos moradores em programas de exercício físico devem estar envolvidos na maior chance de o adolescente ser sedentário em um determinado local.

De fato, destaca-se a importância de espaços públicos adequados e políticas públicas para a promoção da atividade física e a mudança de hábitos da população^29^. Corroborando essa afirmativa, estudo realizado em São Paulo mostrou que a presença de dois ou mais tipos de locais de lazer próximos às casas da população está associada a uma possibilidade 65% maior de realizar caminhadas (OR = 1.65; 95% CI 1.09-2.55)^30^. Portanto, a forma de urbanização dos bairros, considerando ou não espaços apropriados para atividades físicas, devem estar envolvidas com o sedentarismo.

Dessa forma,investimentos em infraestrutura pertinentes à promoção de programas de atividade física são imperativos^31^. Recomenda-se, por exemplo, investimentos do poder público para a adequada implementação de propostas, como o programa Agita São Paulo, com o objetivo de elevar o nível de atividade física da população^33^e o programa Academia da Saúde^34^, criado em 2011, para todas as cidades brasileiras, focando na promoção de exercícios físicos, mudança de hábitos e reeducação alimentar.

Na presente investigação, a prevalência de sedentarismo foi maior em adolescentes do sexo feminino. Esse dado corrobora a Pesquisa Nacional de Saúde do Escolar (PENSE), que reafirma maior prevalência do sedentarismo em meninas(61,3% *versus*58,1%)^5^, assim como o estudo realizado com 899 alunos de ensino médio da rede pública de São José dos Pinhais, Paraná, que evidenciou uma maior razão de prevalência (RP) das meninas relacionadas ao nível insuficientemente ativo (RP: 1,19; IC95%: 1,12-1,27)^32^e com a primeira pesquisa mundial que compara a prática deatividade física e sedentarismo entre as crianças e os adolescentes, realizada pela Organização Mundial da Saúde (OMS). Essa última demonstra que 89% das meninas brasileiras praticam menos exercícios físicos do que o recomendável^35^.

Acredita-se que meninas, desde a fase inicial da adolescência, tendem a participar menos de atividades físicas, especialmente quando possuem menor renda e nível de instrução, pois habitualmente tendem a assumir tarefas domésticas e de trabalho. Além disso, questões tradicionais e culturais de gênero restringem sua participação nas mesmas atividades físicas e esportivas dos meninos^36^. As alterações da puberdade em meninas impactam o desenvolvimento motor, o que pode contribuir para o desinteresse das adolescentes pela realização de esportes^37^. Fica, portanto, clara a necessidade de uma atenção especial neste público com intervenções para promover a atividade física e também investir na criação de espaços onde elas se sintam seguras para praticar esportes.

Por fim, o presente estudo trouxe informações inéditas sobre o sedentarismo e nível socioeconômico em adolescentes da cidade de Divinópolis (MG), levando em consideração os efeitos das diferentes regiões.Há indicadores para que o município desenvolva políticas públicas efetivas no combate ao sedentarismo na população jovem, considerando-se, por exemplo, o plano de ação mundial da Organização Mundial da Saúde (OMS) sobre atividade física e saúde 20182030, que tem como tema: “pessoas mais ativas para um mundo mais saudável”. Esse plano de ação aponta como os países podem diminuir, em 15% até 2030, a inatividade física em adultos e adolescentes, visando criar sociedades mais ativas, por meio da melhoria dos ambientes e oportunidades para os indivíduos de todas as idades se tornarem mais ativos^38^.

Entre possíveis limitações, é importante considerar a natureza transversal do estudo, o que não permite estabelecer uma referência causal entre as variáveis indepentendes e o resultado.

## Conclusão

Maior prevalência de sedentarismo foi identificado entre adolescentes do sexo feminino. O nível socioeconômico e a região onde adolescentes estudam estão associados ao sedentarismo, apontando para a questão da infraestrutura local no favorecimento da realização de atividade física. Esta investigação sinaliza a importância de investimentos públicos em políticas de estímulo à prática de atividade física por adolescentes. Estudos longitudinais sobre a associação entre nível socioeconômico e sedentarismo são indicados para estabelecer a causalidade desse fenômeno.
